# A Simple Chamber for Long-term Confocal Imaging of Root and Hypocotyl Development

**DOI:** 10.3791/55331

**Published:** 2017-05-17

**Authors:** Charlotte Kirchhelle, Ian Moore

**Affiliations:** ^1^Department of Plant Sciences, University of Oxford

**Keywords:** Developmental Biology, Issue 123, Confocal imaging, Plant morphogenesis, Perfluorodecalin, Lateral root development, Time-lapse imaging, dexamethasone, oryzalin, and isoxaben

## Abstract

Several aspects of plant development, such as lateral root morphogenesis, occur on time spans of several days. To study underlying cellular and subcellular processes, high resolution time-lapse microscopy strategies that preserve physiological conditions are required. Plant tissues must have adequate nutrient and water supply with sustained gaseous exchange but, when submerged and immobilized under a coverslip, they are particularly susceptible to anoxia. One strategy that has been successfully employed is the use of a perfusion system to maintain a constant supply of oxygen and nutrients. However, such arrangements can be complicated, cumbersome, and require specialized equipment. Presented here is an alternative strategy for a simple imaging system using perfluorodecalin as an immersion medium. This system is easy to set up, requires minimal equipment, and is easily mounted on a microscope stage, allowing several imaging chambers to be set up and imaged in parallel. In this system, lateral root growth rates are indistinguishable from growth rates under standard conditions on agar plates for the first two days, and lateral root growth continues at reduced rates for at least another day. Plant tissues are supplied with nutrients via an agar slab that can be used also to administer a range of pharmacological compounds. The system was established to monitor lateral root development but is readily adaptable to image other plant organs such as hypocotyls and primary roots.

**Figure:**
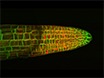


## Introduction

To study cellular and subcellular processes that underlie plant development, there is an increasing demand for high-resolution long-term time-lapse imaging strategies. A key challenge in such imaging experiments is the maintenance of physiological conditions including sufficient gaseous exchange plus a supply of water and nutrients^1^,^2^,^3^. To utilize objectives with high numerical apertures for optimal optical resolution, specimens should be positioned in close proximity and oriented parallel to the coverslip. Movement in x, y, and z directions should ideally be minimal during imaging.

While seedlings are often mounted in water or aqueous solution for short-term imaging, water has a low capacity for dissolving CO_2_ and O_2_ (1.54 mg/mL and 0.04 mg/mL, respectively at 20 °C, 0.1 MPa)^4^, which makes it unsuitable for extended time-lapse experiments. Perfusion systems that maintain constant levels of oxygen and nutrients are one solution to this problem and have been developed for both confocal laser scanning microscopy (CLSM)^1^,^2^,^3^ and light sheet microscopy (LSM)^5^. Systems like the RootChip^2^ and the RootArray^3^ have been designed specifically for time lapse imaging of developing roots and involve germinating seeds in a custom-build multi-specimen device. These arrangements ensure minimal mechanical perturbation and are designed for the parallel analysis of multiple seedlings, but are not optimized for imaging of subcellular structures. Calder and colleagues have designed a more complicated perfusion-based imaging chamber optimized for imaging of subcellular structures in which the specimen is held in position by a mesh to allow the use of high magnification immersion lenses^1^.

Presented here is an alternative, simple solution to this problem which is not based on perfusion systems, but utilizes perfluorodecalin (PFD), a perfluorocarbon that has recently gained popularity as a mounting medium for *Arabidopsis *imaging^6^,^7^,^8^. In such applications, the high capacity of PFD for dissolving CO_2_ and O_2_ (1.9 g/mL for O_2_ in PFD compared to 0.04 mg/mL in water)^9^, allows gaseous exchange by the immersed tissue. Furthermore, PFD is non-fluorescent and its refractive index (1.313) is comparable with that of water (1.333) and is closer to that of cytosol (~1.4) than air (1.000)^6^. Perfluorocarbons have been reported to have minimal physiological effect on a variety of plants and plant tissues^6^, with radish seeds germinating readily when submersed in PFD and exhibiting normal growth and development for at least two full days when supplied with water^10^. Similar observations have been made for germinating *Arabidopsis* seeds^6^. Based on stimulated Raman scattering to directly image the distribution of PFD in *Arabidopsis* leaf tissues after infiltration, Littlejohn and colleagues conclude that PFD is likely not taken up by living cells^8^. PFD has previously been used predominantly to image aerial tissues, where it significantly increases imaging depth as it readily infiltrates air spaces due to its low surface tension^6^. Here, PFD is adopted for long-term confocal imaging of developing lateral roots. In this configuration, one or more seedlings are placed onto a slab of growth medium solidified with agar and immersed in PFD. The PFD allows gaseous exchange in the imaging chamber, preventing anoxia. PFD is highly volatile so it is retained by a gasket of poly(dimethylsiloxane) gum that also has high gas permeability (1.5 x 10^-12^ pmol m^-1^ s^-1^ Pa^-1^ for CO_2_)^11^. Nutrients and water are supplied by the slab of medium solidified with agar. At the same time, this agar slab gently presses the root against the coverslip, thus fixing its relative position in the imaging chamber and allowing the use of high-resolution water immersion lenses. Furthermore, the agar slab can be used to administer a range of pharmacological treatments, including dexamethasone, oryzalin, and isoxaben. The imaging chambers can be assembled in large numbers from standard microscopy slides using minimal equipment. The imaging chambers were developed and characterized to study lateral root development but are adaptable to imaging other seedling organs such as primary root tips and hypocotyls.

## Protocol

### 1. Creating the Chamber

1. Using a glass cutter, cut 3 mm wide strips from the end of a 1 mm thick microscope slide. Using a cyanoacrylate super-glue or double-sided tape, glue these glass strips approximately 45 mm apart across the width of a second microscope slide (Figure 1A). One slide per chamber is required, plus additional slides to pour agar slabs (one slide will yield 2-3 agar slabs). Slides can be reused.
2. **Between the glass strips, fashion a gasket of identical height out of gas-permeant poly(dimethylsiloxane) gum. Place a ball of poly(dimethylsiloxane) gum onto the slide (Figure 1B), wet a second slide with a small quantity of 100% absolute ethanol, and flatten the poly(dimethylsiloxane) gum ball with the second slide until it has reached the height of the glass strips (Figure 1C).**
  1. If necessary, trim excess poly(dimethylsiloxane) gum with a razor blade and repeat flattening.
3. Using a suitable cutter or razor blade wetted in absolute ethanol, remove the interior part of the poly(dimethylsiloxane) gum to create the cavity that will later hold the seedling and agar slab (Figure 1D).
4. Carefully trim the gel with a razor blade to create a gasket with a final wall thickness of approximately 2 mm (Figure 1E). The permeability of gases through a poly(dimethylsilaxane) barrier is independent of thicknesses above 50 µm^11^.

### 2. Creating the Agar-solidified Slab of Growth Medium

1. On a microscopy slide with two glass strips, place a coverslip (22 mm x 50 mm) so that it rests on both glass strips.
2. Pipette melted half-strength Murashige and Skoog growth medium containing 1% w/v sucrose and 1.5% w/v agar (½ MS) into the resulting space below the coverslip until the latter is completely filled (approximately 1 mL total volume) and leave until agar is set (approximately 5 min). Note: Pharmacological compounds can be administered through the agar slab by adding appropriate concentrations to the liquid medium before the slab is poured. This was successfully tested for dexamethasone^12^, isoxaben, 2,6-dichlorobenzonitrile (DCB), oryzalin, and latrunculin B.

### 3*. *Finishing the Chamber Setup

1. Air equilibrate PFD by shaking a small volume of PFD in a tube^7^. Add a small amount (approximately 200 µL) of air-equilibrated PFD to the well of the gel gasket, but do not fill the chamber completely. This PFD will help to avoid the trapping of air bubbles under the agar slab as it is placed into the chamber.
2. Remove the coverslip from the agar slab (at step 2.2 above) and use a razor blade to cut off a portion of desired size and shape. Then place it into the well of the gel gasket (Figure 1F). There should be a gap of 2-4 mm to the gasket all around.
3. Fill the chamber completely with air-equilibrated PFD.
4. Place one or more *A. thaliana *seedlings (up to 3 seedlings for imaging over 2-3 days) onto the agar slab with the cotyledons and hypocotyl hanging over the edge, floating in PFD (Figure 1G).
5. To obtain seedlings, sterilize seeds with 70% ethanol, plant on ½ MS medium supplemented with 1% sucrose and 0.8% agar, stratify for 2-4 days at 4 °C, and grow on vertically oriented plates at 22 °C in a 16 h light/8 h dark regime. For imaging lateral roots, grow plants for 7-10 days before transferring to imaging chambers.
6. To close the chamber, apply a coverslip matched to the optics of the objective, gently pressing it down with the edge of a glass microscope slide until the coverslip rests on both glass strips (Figure 1H).
7. Secure the coverslip with strips of 1.25 cm wide micropore surgical tape cut in half length-wise on each end if desired (Figure 1I). Allow the specimen to settle for approximately 30 min before imaging. This minimizes specimen movement during imaging.
8. Perform imaging with an upright microscope to maintain a controlled substrate for growth. Use 20X/0.7NA or 63X/1.2NA CS2 objectives.

## Representative Results

**Lateral roots grow at physiological rates in imaging chambers.**

Lateral root lengths of plants in imaging chambers were measured at hourly intervals (Figure 2A, left, Movie 1, n = 23) and growth rates were compared to those of lateral roots grown on standard Petri plates containing the same agar-solidified medium (Figure 2A, right, Movie 2, n = 23). Growth rates can vary considerably between lateral roots, with the length of the root being one determining factor owing to the increasingly longer zones of growth and proliferation^13^. Therefore, sets of lateral roots were selected to represent roots of different initial lengths (ranging from 50 µm to 1150 µm) in both the imaging chamber and the agar plate. Average lateral root length at the start of the experiment was similar in each set (439 and 442 µm for imaging chambers and plates, respectively). In the analyzed time interval of 27 h, lateral root growth was approximately linear both in imaging chambers and on plates, with an average growth rate of 52 µm/h (R^2^ = 0.997) on plates and 51 µm/h in imaging chambers (R^2^ = 0.993) (Figure 2B). Growth rates of individual lateral roots where highly variable both on plates and in imaging chambers (Figure 2C), ranging from 17 µm/h to 82 µm/h in imaging chambers and from 13 µm/h to 87 µm/h on plates. While growth rates generally increased with the length of the root, growth rates still varied substantially between roots of similar length but the variance was similar in imaging chambers and on plates.

**Lateral roots proliferate in imaging chambers and can be imaged using high numerical-aperture objectives.**

Lateral roots co-expressing the plasma-membrane marker NPSN12-YFP^14^ and the microtubule marker UBQ1::mRFP-TUBULIN BETA6 (RFP-TUB6)^15^ were imaged by CLSM^10^ at 1 h intervals to document microtubule behavior during cell proliferation in imaging chambers (Figure 2D, Movie 3). In these chambers, lateral roots were highly stable in their position on the x, y and z axes, allowing the acquisition of continuous time-lapse confocal stacks over several hours. Meristematic cells continuously proliferated, as is evident from the formation of preprophase bands (Figure 2D, green arrows), mitotic spindles (Figure 2D, white arrows), and phragmoplasts (Figure 2D, blue arrows). As with seedlings on Petri plates, fully elongated root cells in imaging chambers initiated root hairs (Figure 2A, Figure 2D, arrow heads), indicating further that development proceeded as expected in the imaging chambers. To provide a wide field-of-view, the images in Figure 2D were obtained with a 20x/0.7 NA objective. It was also possible to obtain high resolution images of lateral roots using a high numerical-aperture 63x/1.2 NA water-immersion objective (Figure 2E).

**Lateral root growth is sustained in imaging chambers for up to three days.**

To explore how long imaging chambers could support lateral root growth at physiological rates, lateral root length in imaging chambers (n = 27) and on plates (n = 33) was quantified at 0 h, 24 h, 48 h, and 72 h. The roots chosen were shorter than 200 µm at 0 h, with an average lateral root length of 117 µm in imaging chambers and 121 µm on plates. Since shorter roots grow more slowly on average (Figure 2C), they grew over the edge of the agar slab less often, and were easier to image. The average growth of all lateral roots was not significantly different in imaging chambers compared to Petri plates in the first 48 h (Figure 3A). However, the average growth was significantly reduced between 48 h and 72 h (Figure 3A). While growth rates appear to generally slow down in imaging chambers after 48 h, growth rates between individuals remain highly variable (Figure 3B, **3C**), which means that there is a substantial subset of lateral roots that grows at comparable rates on plates and in chambers over the complete 72 h growth period. 23 of the 33 lateral roots grown on plates (69.7%) reach a length within one standard deviation of the mean length at 72 h (between 927 and 3,163 µm, Figure 3B, red). 10 of 27 (37.0%) lateral roots in imaging chambers reach a length within this interval (Figure 3B, red).

**Imaging chambers can easily be adapted for older lateral roots, primary roots and hypocotyls**.

One of the limitations of the original imaging chamber design was that while the stiff agar slab provided some mechanical resistance, gravitropically growing roots eventually penetrated the agar slab, resulting in loss of image quality (Figure 4B) and growth beyond the working distance of high NA lenses, even the relatively long working-distance (0.3 mm) 63X/1.3 NA CS2 objective. Young lateral roots lack the statolith-containing columella cells required for gravitropism^17^ so they initially grew parallel to the coverslip in imaging chambers (Figure 2E). As they grow longer and columella and statoliths form, lateral roots exhibit increasing positive gravitropism, while primary roots exhibit a strong gravitropic response from germination onwards. This restricts to a few hours the period over which primary roots can be imaged and it severely limits the starting-length of lateral roots that can be imaged continuously for 48 h or more. To overcome this limitation, the chamber was modified such that growth is maintained parallel to the coverslip by a cellulosic cellophane membrane that acts as a penetration barrier on the surface of the agar slab (Figure 4A). Initial attempts using a 1.5% agar slab caused reduced root growth within the first 24 h, owing perhaps to mechanical stress. Lower agar concentrations in the slab were tested and it was found that in chambers with a slab containing 0.8% agar and cellulose film, young lateral root growth rates did not differ significantly from growth rates in conventional chambers for the first 48 h. For 0-24 h, mean root growth in cellulose chambers and conventional chambers was 242 µm and 262 µm respectively (p = 0.78 Student's t-Test) and for 24-48 h was 330 µm and 355 µm^1^ respectively (p = 0.67 Student's t-Test); n = 12 and n = 11, respectively. This arrangement effectively prevented lateral roots from growing away from the coverslip, into the agar, and also allowed imaging of primary roots for up to 48 h (Figure 4C).

To test whether the imaging chambers could be adapted for organs other than roots, *Arabidopsis *hypocotyls were chosen. Hypocotyls are substantially thicker than roots so in the conventional imaging chamber, the uppermost cells were squeezed against the coverslip, leading to deformation. An alternative design was therefore developed using cavity slides that contain an oval depression in their center (Figure 4D). In this configuration, a 1 mm thick slab of agar (prepared as in 2.2 above) was positioned with one end protruding by a few mm into the slide cavity (Figure 4D). Hypocotyls were positioned above the cavity in the slide while the root was positioned above the horizontal part. This ensured that the seedling was fixed in space by the root, but the hypocotyl was not squashed. While growth rates in chambers compared to plates were not systematically quantified, hypocotyls in imaging chambers exhibited the well-described wave of longitudinal growth migrating up the hypocotyl (Figure 4E, **4F**)^16^.

**Figure:**
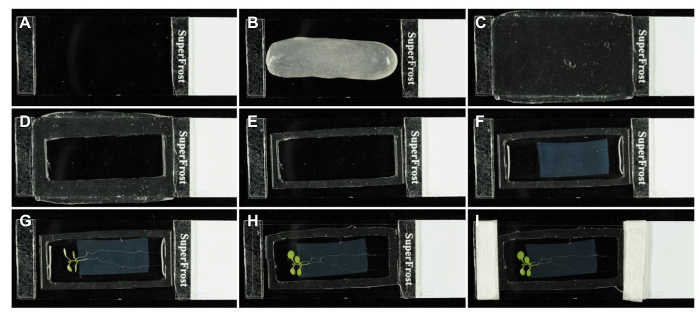

Figure 1**: Assembling an imaging chamber. **All details are described in the text. Please click here to view a larger version of this figure.

**Figure:**
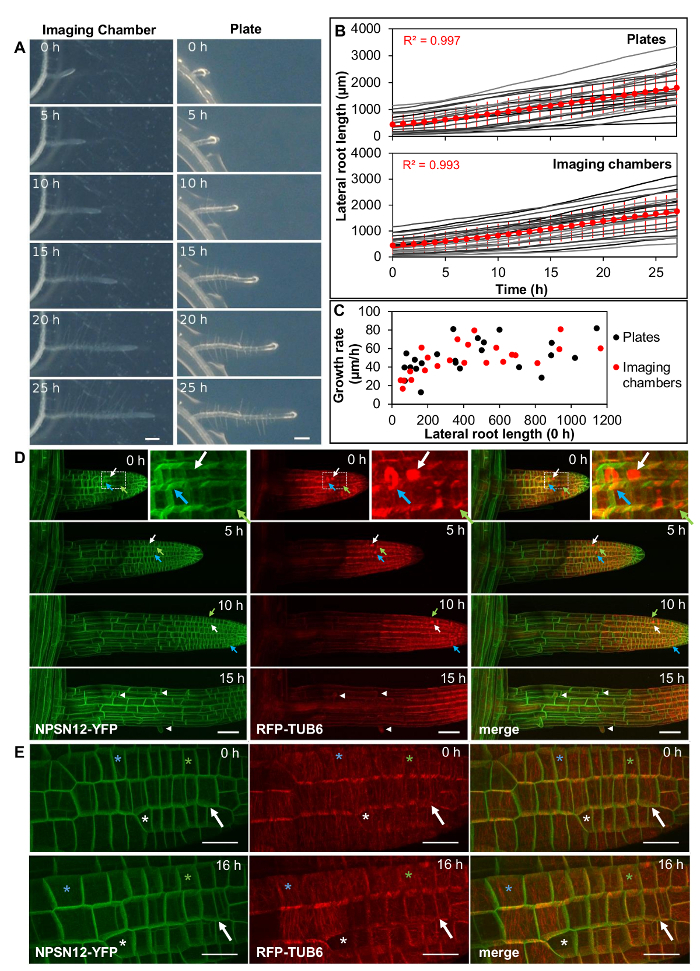

Figure 2**: Lateral roots grow under physiological conditions in imaging chambers. **(**A**)* Arabidopsis* lateral root development in an imaging chamber (left) and on a plate (right) over 25 h (constant light, horizontal incubation, simultaneous imaging). Scale bars = 200 µm. (**B**) Plot showing lateral root lengths over 27 h (measured in 1 h intervals), of lateral roots as shown in (**A**). Individual roots plotted in grey (n = 23 for plates and imaging chambers), average length as red circles. Error bars = 1 SD. Red line is linear fit through average lengths. (**C**) Plot showing average growth rate for all lateral roots shown in (**B**) relative to their initial length. (**D**) Maximum intensity projections of 3D confocal stacks acquired from lateral roots expressing NPSN12-YFP and RFP-TUB6 at consecutive time points. Inset: Magnification of boxed region. Green arrows: preprophase bands; white arrows: mitotic spindles; blue arrows: phragmoplasts; arrowheads: root hairs. (**E**) Maximum intensity projections of high-resolution images acquired from a young lateral root of the transgenic line shown in (**D**) using a 63X/1.2 NA objective and Nyquist sampling (pixel dimensions 77 nm x 77 nm) at 0 h and 16 h; asterisks locate identical cells at each time point; arrow indicates a cell that divides between 0h and 16 h. Scale bars = 50 µm (A-C); 20 µm (E). Please click here to view a larger version of this figure.

**Figure:**
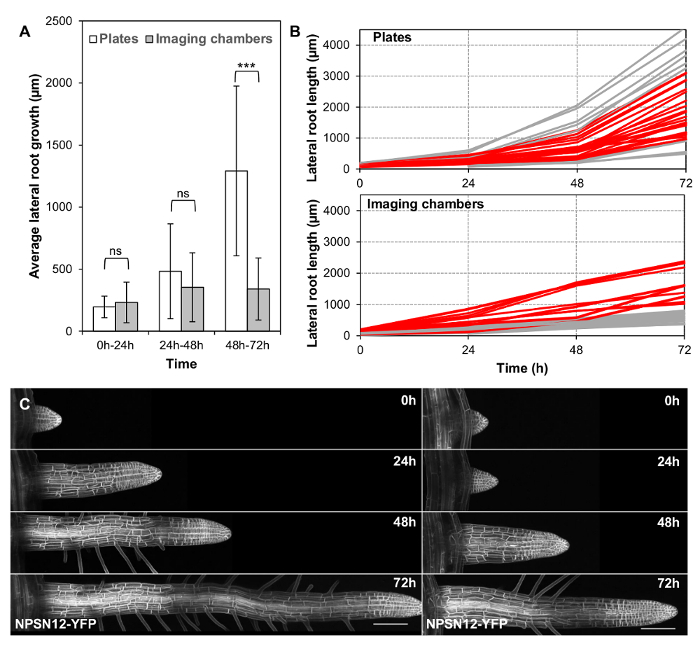

Figure 3**: Long-term lateral root development in imaging chambers. **(**A**) Plot showing average absolute root growth in three consecutive 24 h intervals in imaging chambers compared to plates. n.s. indicates p >0.05; *** indicates p <0.001 (student t-test). n = 33 (plates) and n = 27 (imaging chambers). (**B**) Plot showing lateral root lengths at consecutive time points on all roots used in (A). Red: all lateral roots with a final length within 1 SD of the mean of all lateral roots grown on plates (between 927 and 3163 µm). (**C**) Maximum intensity projections of representative 3D confocal stacks of the plasma-membrane marker NPSN12-YFP in lateral roots, acquired at consecutive time points over 72 h. Scale bars = 100 µm. Please click here to view a larger version of this figure.

**Figure:**
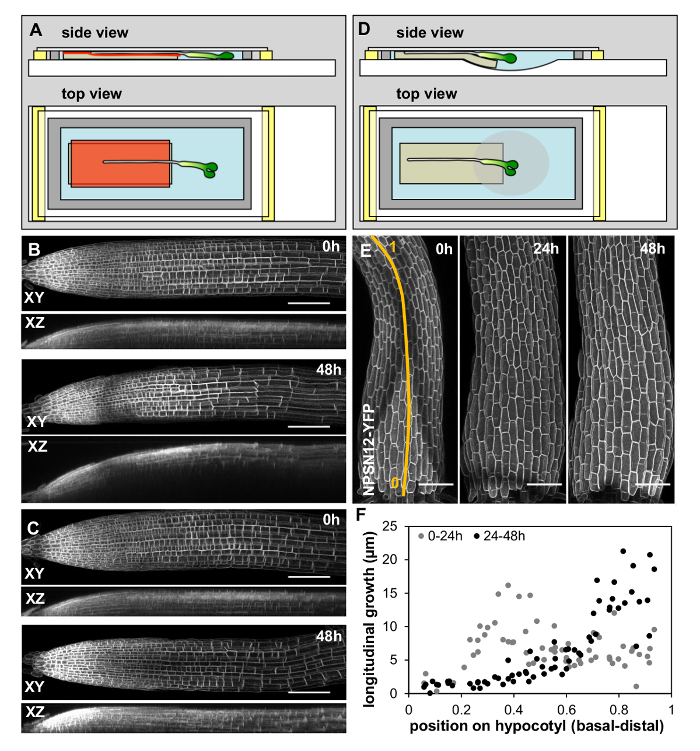

Figure 4**: Imaging chambers can be adapted for other plant organs. **(**A**-**C**) Imaging chamber adaption for primary roots. (**A**) Schematic of imaging chamber design. This is identical to the standard design (Figure 1) apart from two modifications: the MS medium slab contains 0.8% agar instead of 1.5% agar, and a cellulose film (red) is placed between agar and plant to prevent growth into the agar. (**B**) XY (top) and XZ (bottom) maximum intensity projections of representative 3D confocal stacks of the plasma-membrane marker NPSN12-YFP in the primary root of a 7 day old seedling imaged at 0 h and 48 h in a conventional imaging chamber. Note that the root grows into the agar due to its gravitropic response. (**C**) XY (top) and XZ (bottom) maximum intensity projections of representative 3D confocal stacks of the plasma-membrane marker NPSN12-YFP in the primary root of a seven-day old seedling imaged at 0 h and 48 h in the imaging chamber with cellulose film. Before application, the cellulose film was sterilized in 80% ethanol and soaked in liquid ½ MS medium. Note that gravitropic growth of the root into the agar is prevented. (**D**-**F**) Imaging chamber adaptation for hypocotyls. (**D**) Schematic showing hypocotyl imaging chamber design. A poly(dimethylsiloxane) gum gasket (grey) is manufactured on a cavity slide between two glass strips (yellow). A slab of 1.5% agar of even thickness (beige) is placed on the slide, partially reaching into the cavity. The chamber is filled with PFD (blue). Seedlings are placed onto the agar slab so that the hypocotyl occupies the downwards-sloping region of the agar slab in the cavity while the root is positioned the horizontal part of the agar. The chamber is closed with a coverslip. (**E**) Maximum intensity projections of representative 3D confocal stacks of the plasma-membrane marker NPSN12-YFP in a hypocotyl of a two-day old seedling imaged over 48 h at consecutive time points. (**F**) Longitudinal growth in two consecutive time intervals of individual cells along the basal to apical axis (relative positions at 0 h along the axis shown in E) of the hypocotyl shown in (**E**). Note the previously described wave of growth migrating up the hypocotyl^16^. Scale bars = 100 µm. Please click here to view a larger version of this figure.


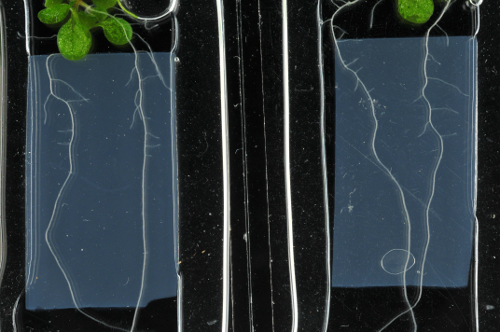
 **Movie 1:** Please click here to download this movie.


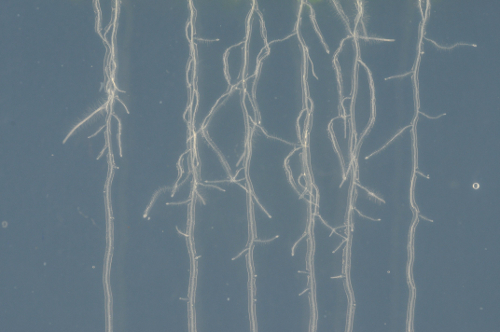
 **Movie 2:** Please click here to download this movie.


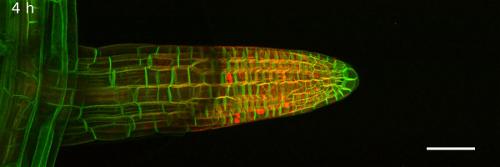
 **Movie 3:** Please click here to download this movie.

## Discussion

The method presented here is a simple strategy for high-resolution confocal imaging of developing lateral roots for two and up to three days. For periods of up to 48 h, no adverse effects of the imaging system on lateral root development were observed. After 48 h, the average lateral root growth began to slow, although a substantial subset of the roots (37%) continued to grow at rates comparable to average root growth on plates. Therefore, through imaging a sufficiently large number of roots, roots whose growth slows after 48 h can be excluded. Systematic tests of the imaging chambers were not performed for periods longer than 72 h, but alternative strategies are recommended if extended imaging periods are desired. Imaging chambers may be left on the microscope stage continuously, if suitable environmental conditions are provided, or removed to a growth facility and periodically returned to the microscope. This allows numerous chambers to be imaged in parallel.

One advantage of the chambers described here is that lateral roots are fixed in their position and can be imaged using high-resolution water immersion lenses. The spatial stability critically depends on the agar concentration used in the supporting agar slab. Initially a range of different concentrations from 0.8% agar to 2% agar were tested, revealing that high concentrations in this range stably fixed roots in space, but root growth slowed down faster and some roots exhibited signs of mechanical stress, including reduced cell elongation. By contrast, low agar concentrations did not provide the required support and roots drifted in x, y, and z during the imaging. The optimal 1.5% agar slab fixes the position of the specimen without adverse mechanical effects. Under these conditions, after settling in the first 30 min or so, roots are stable over many hours, permitting overnight data acquisition. During acquisition of 4D data, z-stack ranges were typically bracketed by an additional 5-10 µm but this was principally to accommodate out-of-plane growth of lateral roots rather than z-drift or wobble. Although the standard agar concentration provides some resistance against penetration, gravitropically growing roots will eventually penetrate the agar. However, through a minor modification of the imaging chamber, root growth could be maintained parallel to the coverslip, allowing older lateral roots and primary roots to be imaged. Furthermore, the basic imaging chamber could easily be customized for hypocotyls. Hypocotyls are more freely floating in this system so the z-axis bracket for image acquisition was increased usually to about 20 µm. In this study, an upright microscope was used throughout, which allowed the substrate properties to be controlled. The imaging chamber may be adaptable to inverted microscope configurations but the time-dependent influence of the rigid coverslip on intercepting organs will need to be evaluated.

Littlejohn and colleagues have pointed out that PFD itself does not readily dissolve biological molecules, which means it cannot be used for the directly for the delivery of pharmacological compounds^7^. This problem was overcome by supplying such compounds through the slab of solidified growth medium on which the agar slab rests. While perfusion systems will remain the method of choice for wash-out experiments, the imaging chamber has been used successfully for the administration of dexamethasone^12^ and other compounds. One note, while this article was in preparation von Wagenheim and colleagues^18^ described a chamber for imaging lateral root development using light-sheet microscopy.

## Disclosures

The authors have nothing to disclose.
